# Extirpation of the Tonsil

**Published:** 1829-07-01

**Authors:** 


### XXIX.

### EXTIRPATION OF THE TONSIL.

The following case, which is related by Dr. Auchincloss, one of the surgeons of the Glasgow Infirmary, in the 5th number of the Glasgow Journal, deserves to be put on record, as it illustrates a homely, but practical point in surgery.

William M'Innes, 39 years of age, applied to Dr. Auchincloss, on the 7th November, to have an operation performed on his left tonsil. This occupied so completely the posterior fauces, that difficulty was experienced in passing the finger between it and the walls of the throat. It had a broad fleshy base by which it was attached to the upper part of the pharynx; its surface was of bluish dirty hue, and lobulated with ulceration in the interstices of the lobules; it was firm to the touch, save at one point, which was indistinctly elastic; the uvula was pushed to the roof of the mouth, but the neighbouring parts appeared healthy. He had no pain in the tonsil when pressed, and only complained of difficuly of breathing and of deglutition. The tonsil, he said, had begun to swell 18 months before, after a syphilitic affection of the throat, for the cure of which he used a great deal of mercury. It produced but little inconvenience till about two months before his application, since which it had rapidly increased in size. He had fallen off much in flesh and strength, particularly within the preceding fortnight.

On the following day, assisted by Dr. Young, and in the presence of several respectable practitioners, Dr. Auchincloss extirpated the tumour with a curved probe-pointed bistoury. Having previously seized it by the middle with a pair of double forked forceps, by which it was pulled towards the opposite side by the assistant, he introduced the bistoury to its lower part along his fore-finger, with which he depressed the tongue, and cut from below two thirds up, on a line with the edge of the anterior pillar of the velum. The knife was then withdrawn, and seizing the forceps, the section was completed from above downwards. Scarcely any bleeding ensued, the throat was gargled frequently with a strong solution of alum; on the 10th, the wound was rubbed with the lunar caustic; on the 7th day after the operation, the wound had cicatrized, and the patient is at present perfectly well. The part of the tonsil removed measured three inches by an inch and a half in its lesser diameter, and on cutting it open, presented a variety of structure. It was dense and firmly organized around its circumference, but partly fibrous, and partly spongy, with a number of small cysts towards the centre. In some of these cysts was contained a fluid somewhat resembling putrid blood, and in others a gelatinous semi-organized matter. A small quantity of pus had escaped during the operation; the base along the cut surface was soft, and in every other respect seemed free from disease.

"Two practical inferences may be deduced from the foregoing case. 1st, That excision of an enlarged tonsil may be had recourse to with safety, in so far as hæmorrhage is concerned; and 2d, that however much enlarged, or otherwise of a suspicious appearance, it does not necessarily follow that the disease is malignant, and it may therefore be partly removed without the least risk of the remainder assuming a bad character.

"It would certainly be improper to generalize from any individual case, but I should conceive that there are a sufficient number of instances on record to prove the correctness of what is now alleged. Bertrandi, and several others, are said to have repeatedly extirpated enlarged tonsils, and to have never met with an instance of danger from bleeding. Dupuytren, at his *Clinique Externe*, is in the frequent habit of doing the same operation, without ever dreading hæmorrhage.

"As already stated, the exterior of the tumour prior to the operation, and its appearance on section after removal, seemed to indicate a malignant tendency. In this, however, we were deceived, by the part cicatrizing in the course of a few days—a sufficient proof that the disease was not of a cancerous or otherwise serious nature. By some authors this chronic enlargement of the tonsil has been improperly termed schirrhus. With many it is still a matter of doubt, whether or not cancer ever affects this part of the body. We are informed by Boyer that instances of this kind have occurred, but they are at least exceedingly rare, and perhaps it may be fairly doubted whether such an affection ever exists.

"Of the two modes of removing enlarged tonsils, by the knife and the ligature, I should be disposed, in every case, to prefer the former, not only because it is the more expeditious, but, in reality, it appears to me fully the safer.

"I may mention, that in the above case, it was recommended to me to make use of the ligature, from an idea that its section might be attended with dangerous hæmorrhage. Considering the great bulk of the tumour, it was presumed, that the nutrient vessels would be very numerous, and in a state of enlargement. Had the ligature been used, I should at the same time have deemed it proper to cut off a part, so as to obviate any risk of suffocation from the increased bulk of the strangulated tumour."

We cannot lay much stress on cicatrization of the wound, as proving that the disease was not malignant. We do not believe that every malignant tumour cut into or cut across must necessarily pass into the state of ulceration, indeed we know that such is not the case. At the same time we allow that, most probably, the enlargement of the tonsil in the present instance had not a genuine malignant nature, because such disease in such a situation is certainly very uncommon. For the most part the enlargements of the tonsil which follow the venereal or mercurial poisons, or occur in scrofulous subjects, present no particular change of structure. With regard to the mode of operation we fully agree in all that has fallen from the author of the case. We have frequently witnessed tonsils of considerable size removed by the curved probe-pointed scissors, and never with any ill consequences. Mr. Brodie is constantly performing the operation, and in no instance has he met with serious hæmorrhage. In children, Mr. Brodie takes hold of the tonsil with the Assalini's tenaculum, and snips off more or less of the gland with the curved probe-pointed scissors. The inconveniences of the ligature are very great,—those of excision trifling in practice, and greatly over-rated in books.

